# Ultrasound Imaging of Head/Neck Muscles and Their Fasciae: An Observational Study

**DOI:** 10.3389/fresc.2021.743553

**Published:** 2021-12-15

**Authors:** Carmelo Pirri, Caterina Fede, Chenglei Fan, Diego Guidolin, Veronica Macchi, Raffaele De Caro, Carla Stecco

**Affiliations:** Department of Neurosciences, Institute of Human Anatomy, University of Padova, Padova, Italy

**Keywords:** masticatory muscles, temporal muscle, deep fascia, ultrasonography, masseter muscle, sternocleidomastoid muscle

## Abstract

**Background:** Masticatory muscle thickness provides objective measurements of the temporomandibular motor function, which may change in patients with oral myofascial pain. Moreover, they are considered as being part of the craniocervical unit by a crucial relationship with cervical muscles and their fasciae. In this study, we aimed to assess by ultrasound (US) imaging the fasciae of the masseter, temporal, and sternocleidomastoid muscles to understand their mean thickness and eventual variation in relationship with the muscles, sides, and sex.

**Methods:** We studied 16 healthy volunteers without temporomandibular joint dysfunction. Concerning each subject were evaluated the range of motion of the temporomandibular joint and of the neck, the thickness of muscles and their fasciae of both sides, and the delta of muscle thickness.

**Results:** All the motor evaluations of the subjects showed normal ranges. The US results showed that the fasciae have a mean thickness of 0.50 ± 0.1 mm, which did not change during muscle contraction. The evaluated muscles presented a symmetry between right and left (*p* > 0.05), even if the delta of muscle (US) thickness had a huge range between different subjects, for example in the masseter muscle from 0.7 to 4.2 mm.

**Conclusions:** Ultrasound imaging is a suitable and reliable tool to study the muscles and fasciae of the head and neck region, permitting also the evaluation of the ability of the muscles to contract. Finally, identifying functional asymmetry that could become symptomatic, US imaging could allow an early rehabilitation treatment.

## Introduction

The masticatory muscles have a crucial role in the control of the position and motion of the mandible, creating forces at the teeth and temporomandibular joint (TMJ) (1). The TMJ disorders are intricate dysfunctions involving the masticatory muscles and TMJ. The etiology of this dysfunction is usually imputed to a parafunctional activity in the stomatognathic system (2). The scientific literature is strong and consistent to support the role of different factors such as psychosocial, genetic issues, and muscle-related overload, in the pathophysiology of TMJ disorders (3, 4). Intensive use and prolonged high activity of these muscles manifest in an increase of the ultrasonographic thickness of the masseter muscle and increased maximal bite force values (5). Various imaging modalities, such as CT, MRI, and ultrasound (US) were used to visualize and assess the masticatory muscle (6, 7). Among these techniques, US imaging is a method that has been demonstrated to be able of giving information by assessing muscle structural alterations (8), ensuring better clinical availability and costs (9). Masticatory muscles thickness provides objective measurements of the temporomandibular motor function, which may change in patients with oral myofascial pain. Moreover, they are considered as being part of the craniocervical unit by a crucial relationship with cervical muscles and their fasciae (10). Although, some studies (7, 11, 12) investigated only the masticatory muscles thickness by US imaging, but no study assessed the US fasciae thickness of these muscles and of those head and cervical muscles that are related to them. Chang PH et al. (7), for example reported in a population of 48 healthy volunteers, a mean US thickness for the temporalis of about 5 mm and for the middle masseter of about 14 mm. Moreover, concerning a population of young children, Midori Castelo P et al. (12) reported a masseter muscle thickness ranged from 9.36 to 10.54 mm in the relaxed position, and from 10.92 to 12.17 mm in maximum intercuspation, while concerning temporalis muscle thickness the values ranged from 2.54 to 2.76 and 3.24 to 3.52 mm. In the recent literature, some studies set out to evaluate the US fasciae thickness in other topographical regions (11, 12), demonstrating good reliability in the US measurements (13–15). Therefore, the main purpose of the present study was to bilaterally assess the US fasciae thickness of the masseter, temporal, and sternocleidomastoid (SCOM) muscles and their US muscles thickness, evaluating the symmetry of muscles and, respectively of their fasciae in healthy people. The second aim was to find a relation between the US thickness of muscles and fasciae with the range of motion of the temporomandibular joint and of the neck. The third purpose was to assess the delta muscle thickness of the evaluated muscles. Finally, the fourth purpose was to assess the intra-rater reliability of the US thickness measurements of the fasciae and their muscle.

## Materials and Methods

### Design of the Study

A cross-sectional study based on the Strengthening the Reporting of Observational Studies in Epidemiology (STROBE) statement was conducted (16) in order to compare the US thicknesses of the temporal, masseter, and SCOM muscles and their fasciae. The Helsinki Declaration and human experimentation rules (17) were considered and previously, the Ethics Committee of the University of Padua approved the research. All participants were informed prior to inclusion in the project by providing a written consent form.

### Study Population

This study included 16 healthy subjects (nine female and seven male) aged 22 ± 3.2 years. A visual analog scale was performed, and only the participants with a value of 0 were recruited. Moreover, a medical interview was made to collect detailed information about the current general health conditions. Participants were eligible for the study if they met the following criteria: not experience for: oral myofascial pain, temporomandibular dysfunction (TMD), sleep bruxism, cervical pain, history of facial trauma, any pain, or restrictions in terms of mandibular movement, open bite or a crossbite, a prominent facial asymmetry. Subjects were excluded if they exhibited any of the following: history of TMJ surgery or steroid injections, comorbid fibromyalgia, diagnosis of systematic disease (rheumatoid arthritis, systemic lupus erythematous, and psoriatic arthritis), central and peripheral nervous system diseases, previous cervical or head trauma, diagnosis of primary headache (tension-type headache or migraine), lack of dental or physiotherapeutic treatment, cognitive impairment.

### Sample Size Calculation

According to the previous studies in the literature about the masticatory muscle US thickness, was calculated the sample size by the use of the G power 3.1.9.2. software, based on a previous study investigating the thickness of masseter muscles in patients without temporomandibular joint dysfunction (7). We assumed that a variation among certain demographic factors led to a mean difference of 1.4 mm in muscle thickness with a standard deviation of 0.9 mm. The alpha level was set at 0.05 with a power of 80%. Considering a drop-out rate of 20%, the total number needed was 14. The resulting effect size from our measurements, for the muscles' thickness, was d = 2.28, while concerning the fascia's thickness, the effect size was d = 0.93. The enrolment of the subjects was performed by a specialized medical doctor with more than 5 years of experience in physical and rehabilitation medicine.

### Ultrasound Measurements

Using a high-resolution device (Edge II, Sonosite) with a frequency range of 6–15 MHz and a screen resolution of 1,680 × 1,050 pixels. A physician specialist in Physical and Rehabilitation Medicine with 5 years' experience in skeletal-muscle and fasciae US imaging carried out the US assessments. The US was set to B-mode and depicted a depth of 40 mm. The axial and lateral resolution were 0.1 and 0.2 mm, respectively. The US beam was kept perpendicular to the fascial layers which seem to be prone to anisotropy artifacts. The power and overall gain of the ultrasound machine were adjusted to optimize the visualization of the fascial planes and muscles and to obtain the best possible views and scans (18, 19). Concerning adequate scans and to reduce surface pressure on the skin, the ultrasonographer used suitable amounts of gel. The probe was placed on the skin as lightly as possible to avoid tissue compression but was quite stable to maintain adequate contact between the probe and skin for consistent images.

The muscle thickness was defined as the maximal distance between the outer and inner fasciae. The fasciae appear as echogenic bands upon US imaging, contrasting very well with the surrounding tissues. More specifically, the deep fascia appears as a thin hyperechoic band adhering to the muscle.

The subjects lay in a supine zero position (hands along the body and palms facing upwards, legs slightly apart and feet in slight external rotation), with the head in a neutral position, without contact between the teeth and with the superior and inferior lips in contact with each other, the rest of the body relaxed.

The images are taken directly during the US assessment, in real-time, and the measurements are made simultaneously with the examination.

**Figure 1:**
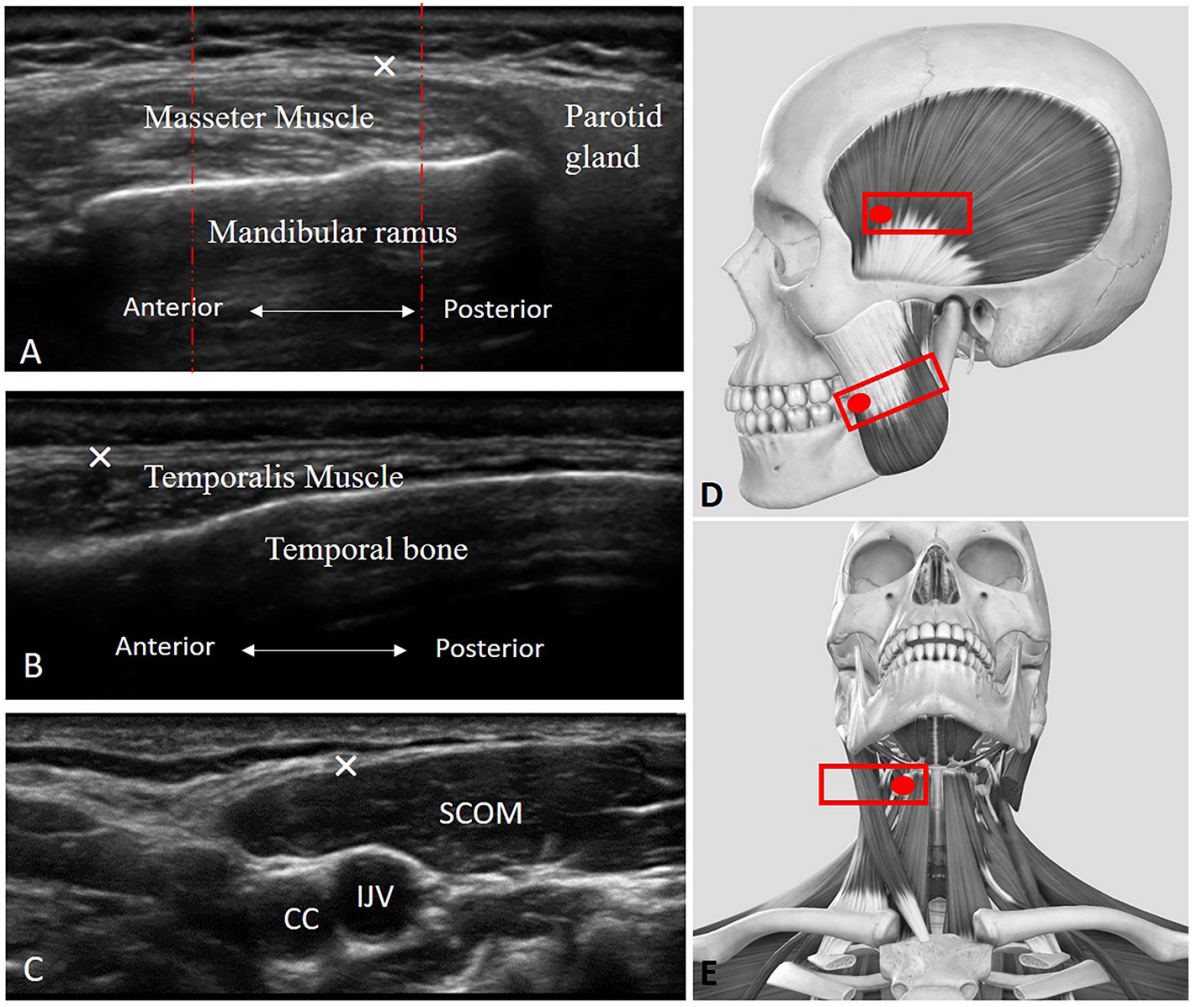
Ultrasound (US) images of the masseter **(A)**, temporalis **(B)**, and scom **(C)** muscles with their fasciae. CC, common carotid artery; IJV, internal jugular vein; SCOM, sternocleidomastoid muscle; ×, deep fasciae. **(D)** Transducer placement for scanning of the masseter and temporalis muscles. **(E)** Transducer placement for scanning of the SCOM muscle. Red dashed lines: show how the three measures were taken in the three equidistant regions per image/structure identified.

The thickness of the middle masseter muscle (Figures 1A,D) was visualized according to Chang KV et al. (7), and the masseteric fascia thickness was visualized, defined, and measured as the maximal distance between the superficial layer and deep layer of the masseteric fascia. Muscle and fasciae thicknesses were bilaterally measured during relaxation and maxima voluntary contraction (maximal jaw clenching).

The thickness of the temporalis muscle (Figures 1B,D) was visualized according to Chang KV et al. (7), and temporal fascia thickness was visualized, defined, and measured as the maximal distance between the superficial and deep layer of the temporal fascia. Muscle and fasciae thicknesses were bilaterally measured during relaxation and maxima voluntary contraction (maximal jaw clenching).

While the thickness of the SCOM muscle was visualized (Figures 1C,E) using the landmark of the carotid artery and internal jugular vein in the middle of the neck, under the mandibular bone. The SCOM fascia thickness was visualized, defined, and measured as the maximal distance between the superficial and the deep layer of the SCOM fascia. Muscle and fascia thicknesses were bilaterally measured during relaxation and maxima voluntary contraction (the subjects were asked to lift their forehead against resistance applied by an operator, just lifting the head from the table, to avoid excessive flexion of the neck).

To eliminate the influence of possible thickness variations, three equidistant points per image/structure were measured and the values were averaged and analyzed. The ultrasonographer followed the protocol carefully to ensure that each point of the muscles and of the fasciae was quantified in the same way.

### Range of Motion of TMJ and Neck Assessments

The range of motion of the TMJ was assessed in the different movements. A millimeter ruler was used to analyze mandibular movements. The volunteers were asked to follow the physician's instructions. The examining physician used the distance between the edges of upper and lower incisors, by the following protocol:

- Lateral movement: the subject is seated and the dorsal-lumbar spine resting on the backrest with the head in position 0, the measurement is made by placing the millimeter ruler on the upper incisors. The midline between mandibular central incisors was overlap with the position number 5 cm on the ruler. The range of lateral movements was determined by moving the mandible to the left and right against that reference point. The volunteers were instructed to move the mandible only in the lateral plane instead of moving the mandible anteriorly and laterally, which is frequently a mistake.
- Opening of the mouth (depression): the subject is seated and the dorsal-lumbar spine resting on the backrest and the head in position 0. Starting from point 0, the downward movement of the mandible is measured by placing the ruler on the edges of the lower incisors. The range of mandible depression is measured along the mandibular midline.

Placed on the upper edge of the upper lip with respect to the lower lip file lower with the mouth at maximum opening in a central position in line with the sub-nasal fossa.

- Protrusion: the subject seated and the dorsal-lumbar spine resting on the backrest and the head in position 0. The next step was to measure the level of protraction from the centrical occlusal position to the maximum forward movement of the mandible. The protraction was measured at a minimal distance between the upper and lower teeth.
- Retrusion: the subject seated and the back-lumbar spine resting on the backrest and the head in position 0. The next step was to measure the level of retrusion from the centrical occlusal position to the maximum backward movement of the mandible. The retrusion was measured at a minimal distance between the upper and lower teeth.

Moreover, also the range of motion of the cervical spine was assessed in different movements. A goniometer (Arthrodial Goniometer, Baseline® Large Joint Protractor) was used to assess the neck movements:

- Flexion-extension of the head and neck: the subject seated and the back-lumbar spine resting on the backrest, the measurement was made in degrees with the use of a goniometer in which the pin is positioned on the projection of C7 (protractor over the shoulder) fixed arm pointed at the vertex of the head in position 0 and the mobile arm pointed at the vertex of the head when the movement was complete.
- Rotation of the head and neck: with the subject seated with the dorsal-lumbar spine resting on the backrest and the subject is fixed with the upper limbs to the backrest to prevent rotation of the dorsal-lumbar spine. The measurement is made with the goniometer with the pivot on the axis of rotation at the level of the vertex, the fixed arm pointed at the nose with the head in position 0 and the movable arm pointed at the nose when the movement is complete.
- Lateral inclination of the head and neck: with the subject seated and the dorsal-lumbar spine resting on the backrest, the measurement is made in degrees with the goniometer pivot on the rotation axis at the level of C7, the fixed arm pointed at the vertex with the head in position 0 and the movable arm aimed at the vertex when the movement is complete.

### Statistical Analysis

Statistical analysis was performed using GraphPad PRISM 8.4.2 (GraphPad Software Inc., San 180 Diego, CA, USA) and *p* < 0.05 was always considered as the limit for statistical significance. The normality assessment was carried out using the Shapiro–Wilk test or Kolmogorov–Smirnov test and Levene's test was performed to investigate the homogeneity of variance. Descriptive statistics were calculated, such as measures of central tendency and their dispersion ranges using the mean and SD to describe parametric data. A comparative analysis between the opposite ROM was assessed performing paired Student's *t*-test. Differences US-estimated thickness across muscles and across fasciae in relaxed and contracted states between right and left were statistically analyzed by three-way ANOVA followed by Tukey's multiple comparisons test for multiple comparisons. In addition, the Pearson's test was employed to evaluate the correlation between BMI, weight, height, age, and muscles and fasciae. The two-way mixed model intra-class correlation coefficient (ICC_3,1_), type C, was used to evaluate the intra-rater reliability. ICC values were interpreted as poor when below 0.5, as moderate when between 0.5 and 0.75, as good when between 0.75 and 0.90, and as excellent when above 0.90 (19). Ninty five percentage CI are reported parenthetically after the group estimator where applicable. SPSS version 21 was used for all statistical and reliability analyses (SPSS Inc., Chicago, IL, USA).

## Results

### Descriptive Data of the Sample

Characteristics of the 16 healthy volunteers are shown in Table 1. Mean age ± SD of the subjects was 22 ± 3.30 years (range, 20–24), and mean BMI ± SD was 24.7 ± 4.80 Kg/cm^2^.

**Table 1:** Demographic features of the healthy volunteers.

**Subject**	**Sex**	**Age**	**Weight (Kg)**	**Height (cm)**	**BMI (Kg/m^**2**^)**
S1	F	30	60	172	20.3
S2	F	20	81	170	28.03
S3	F	19	64	166	23.2
S4	M	22	94	176	30.4
S5	F	24	61	169	21.4
S6	M	24	105	175	34.3
S7	F	16	60	156	25
S8	F	18	66	162	25.2
S9	M	24	122	193	33
S10	M	26	85	172	29
S11	F	21	55	161	20.2
S12	F	22	61	165	22.4
S13	M	21	65	175	21.2
S14	F	22	47	160	18.4
S15	M	21	65	173	22
S16	M	22	76	184	23
**Mean ± SD**	**9 F + 7 M**	**22 ± 3.3**	**72.94 ± 20**	**170.6 ± 9.3**	**24.70 ± 4.8**

### Range of Motion of TMJ and Neck Assessments

**Figure 2:**
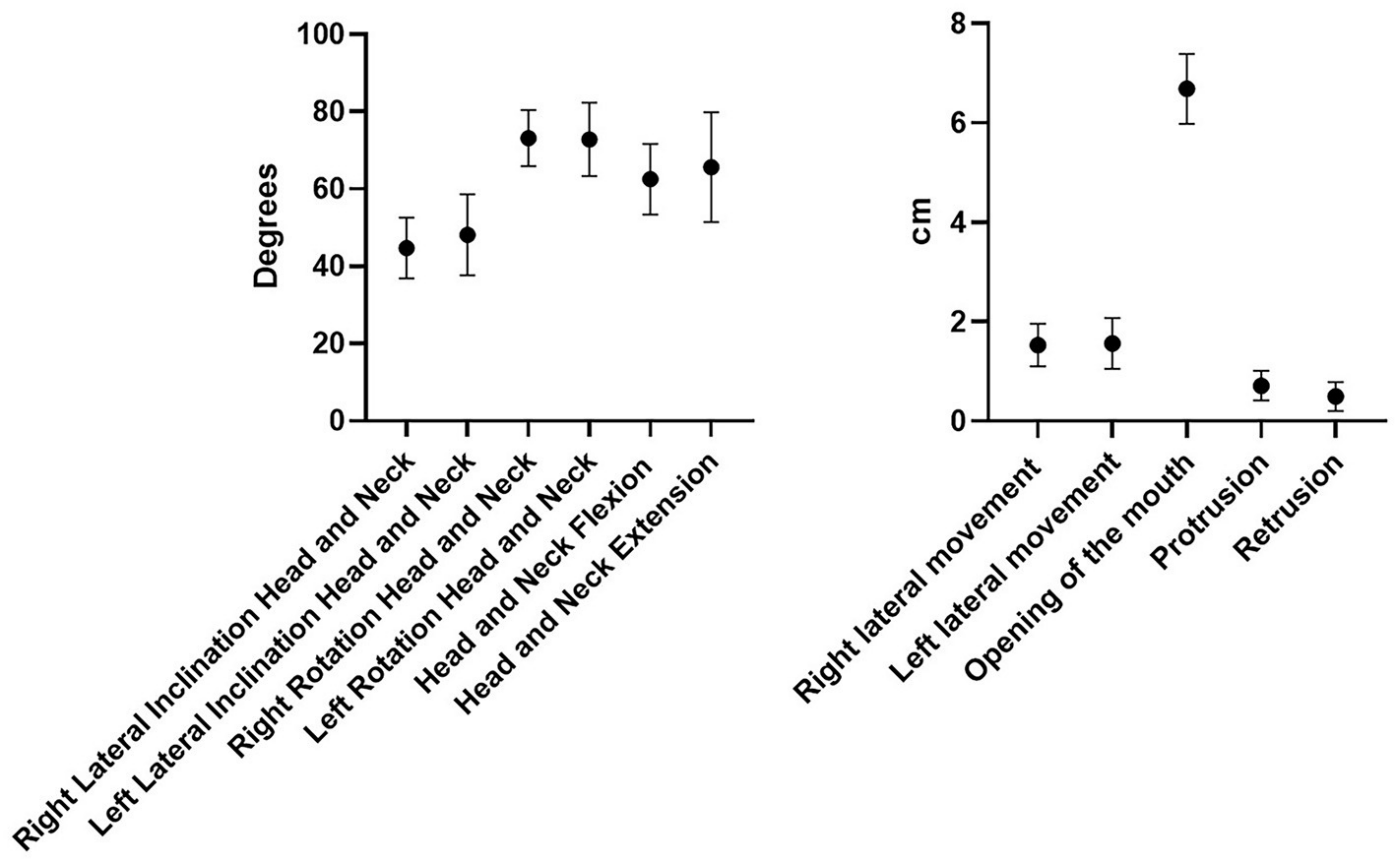
ROM in the different movements of the neck/head and temporomandibular joint.

All the ROM of the chosen movements were considered normal, and the differences among subjects were not statistically significant (*p* > 0.05) except for the retrusion and protrusion of the mandible (*p* < 0.43) (Figure 2).

### Ultrasound Measurements of Muscles Thickness

**Figure 3:**
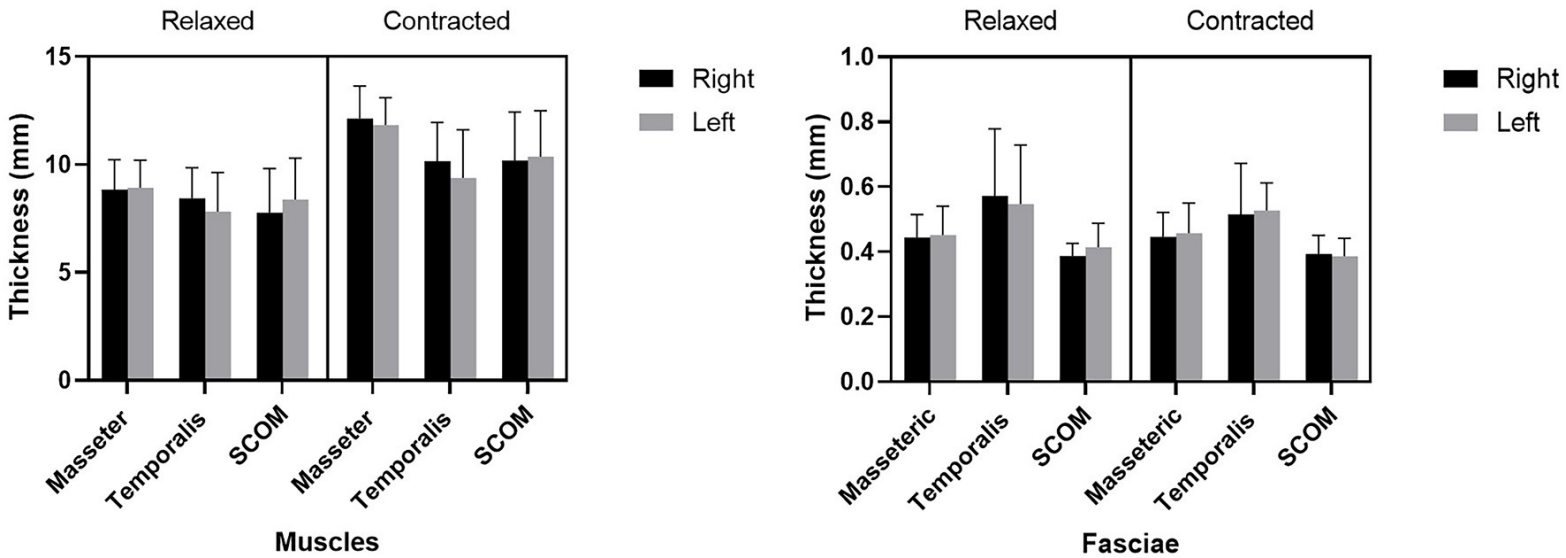
The US thicknesses (mm) of the masseter, temporalis, and sternocleidomastoid (scom) muscles and the US thickness of their fasciae.

**Figure 4:**
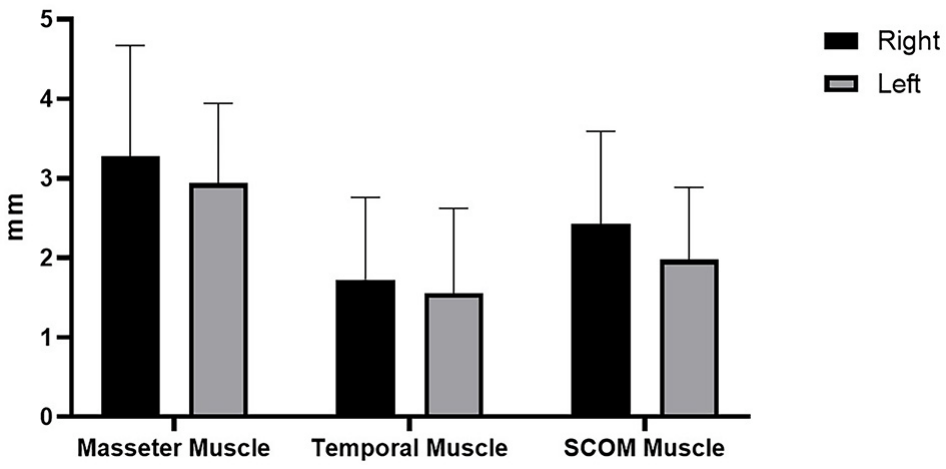
Delta of muscle thickness (between the relaxation phase and the contraction phase) of the right and left muscles' thickness (mm).

Ultrasound thickness of the muscles was respectively in relaxed vs. contracted states, for masseter muscle: 8.90 ± 1.31 mm vs. 11.98 ± 1.40 mm, for temporalis muscle 8.134 ± 1.64 mm vs. 9.80 ± 2.04 mm, for SCOM muscle: 8.10 ± 2 mm vs. 10.28 ± 2.2 mm (Figure 3). When comparing the muscle thickness between both sides, there were no statistically significant differences (*p* > 0.05), even if, evaluating the single subjects, in many cases, a different muscle thickness in one side with respect to the contralateral side was highlighted. The difference in muscle thickness in the relaxed and contracted situation was statistically significant (*p* < 0.001) for all the considered districts. The results obtained showed the symmetry between right and left (*p* > 0.05). The differences of the delta of US muscle thickness between the two sides and among the various participants were not statistically significant (*p* > 0.05) (Figures 3, 4, Tables 2, 3).

**Table 2:** Delta of muscle thickness (between the relaxation phase and the contraction phase) of the right and left muscles' thickness (mm).

**Subject**	**Masseter right**	**Masseter left**	**Temporalis right**	**Temporalis left**	**Scom right**	**Scom left**
S1	4.2	2.9	0.1	0.4	2.4	1.7
S2	4.2	2	2.2	1.7	0.6	1.6
S3	0.5	1.5	0.1	1.7	2.5	2
S4	0.7	1.3	2.3	0.2	1.9	1.9
S5	4.9	2.9	2.6	1.3	1.4	0.3
S6	2.5	2.9	1.2	0.6	3.9	2.5
S7	4.4	4	0	0.2	2	2
S8	5.1	3.9	2.2	2.6	0.8	2.5
S9	3.2	1.8	1.5	0.7	1.6	0.6
S10	4	2.5	1.6	2.2	2	3.5
S11	3.1	3	1.9	3.9	3.2	0.7
S12	2.7	2.7	1	0.8	3.5	2.7
S13	4.7	4.6	1.7	2.1	4.1	3.5
S14	2.5	4.7	2.9	3.1	4.6	2.3
S15	2.1	3.1	3.2	1.9	1.8	2.1
S16	3.7	3.2	3.1	1.5	2.6	1.7
**Mean ± SD**	**3.28 ± 1.4**	**2.93 ± 1**	**1.72 ± 1.03**	**1.55 ± 1.1**	**2.43 ± 1.2**	**1.97 ± 1**

**Table 3:** Comparison between the right side and left side about the delta of muscle thickness.

**Tukey's multiple comparisons test (Right–Left)**	**Mean diff**,	**95,00% CI of diff**	**Significant?**	**Adjusted P value**
Masseter muscle	0.3	−0.6 to 1.2	No	0.638
Temporalis muscle	0.2	−0.7 to 1.1	No	0.9631
SCOM muscle	0.5	−0.5 to 1.4	No	0.5722

### Ultrasound Measurements of Fasciae Thickness

Ultrasound thickness of the fasciae were respectively in relaxed vs. contracted states, for masseteric fascia: 0.45 ± 0.10 mm *vs.*. 0.45 ± 0.08 mm, for temporal fascia: 0.60 ± 0.20 mm vs. 0.52 ± 0.12 mm, for SCOM fascia: 0.40 ± 0.10 mm vs. 0.40 ± 0.10 mm (Figure 3, Tables 4, 5). According to Tukey's multiple comparisons test, the comparison between fascia thickness among various districts showed a statistically significant difference (Figure 2, Table 5), while it does not vary in the relaxed and contracted situation (*p* > 0.05).

**Table 4:** The ultrasound thicknesses (mm) of the masseter, temporalis, and scom muscles in relaxed and contracted phases.

	**Relaxed masseter muscle**	**Contracted masseter muscle**	**Relaxed temporalis muscle**	**Contracted temporalis muscle**	**Relaxed SCOM muscle**	**Contracted SCOM muscle**
Number of values	32	32	32	32	32	32
Minimum	6	9.7	3.8	4.7	4.7	6.4
Maximum	11.6	15.4	11.1	13.1	11.4	14.8
Range	5.6	5.7	7.3	8.4	6.7	8.4
Mean	8.8	11.9	8.1	9.7	8	10.2
Std. deviation	1.3	1.3	1.6	2	1.9	2.1
Std. error of mean	0.2	0.2	0.2	0.3	0.3	0.3
Lower 95% CI of mean	8.4	11.4	7.5	9	7.3	9.5
Upper 95% CI of mean	9.3	12.4	8.7	10.5	8.7	11
Coefficient of variation	14.7%	11.6%	20%	20.8%	24.5%	20.9%

**Table 5:** The ultrasound thicknesses (mm) of the masseteric, temporalis and scom fasciae in relaxed and contracted phases.

	**Relaxed masseteric fascia**	**Contracted masseteric fascia**	**Relaxed temporal fascia**	**Contracted temporal fascia**	**Relaxed SCOM fascia**	**Contracted SCOM fascia**
Number of values	32	32	32	32	32	32
Minimum	0.3	0.3	0.3	0.3	0.3	0.3
Maximum	0.6	0.6	1.3	1	0.5	0.4
Range	0.3	0.3	0.9	0.6	0.2	0.1
Mean	0.5	0.5	0.5	0.5	0.4	0.4
Std. deviation	0.1	0.1	0.2	0.1	0.1	0.1
Std. error of mean	0.01	0.01	0.03	0.02	0.01	0.01
Lower 95% CI of mean	0.4	0.4	0.5	0.5	0.4	0.4
Upper 95% CI of mean	0.5	0.5	0.6	0.6	0.4	0.4
Coefficient of variation	17.6%	18.5%	34.3%	23.7%	14.8%	14.3%

### Correlation Analysis

According to the correlation analysis, there were some statistically significant correlations between muscles and fasciae thickness and age, weight, height, BMI, as reported in Tables 6, 7.

**Table 6:** Correlation (Pearson R coefficient test) between fasciae Ultrasound measurements and Age, Height, Weight, BMI. Only statistically significant data are reported.

**Fascia**	**Data**	**r**	***p*-Value**	**95% CI of diff**
Relaxed SCOM muscle	BMI	0.362	*p =* 0.0418	0.02–0.6
Contracted SCOM muscle	BMI	0.5226	*p* = 0.0022	0.2–0.7
Relaxed Masseter muscle	Weight	0.3878	*p* = 0.0283	0.1–0.7
Contracted SCOM muscle	Weight	0.3912	*p* = 0.0268	0.1–0.7

**Table 7:** Correlation (Pearson R coefficient test) between muscles Ultrasound measurements and Age, Height, Weight, BMI. Only statistically significant data are reported.

**Muscle**	**Data**	**r**	***p*-Value**	**95% CI of diff**
Relaxed masseter muscle	Age	0.4481	*p* = 0.0101	0.1–0.7
Contracted masseter muscle	Age	0.3822	*p* = 0.0309	0.04–0.7
Relaxed temporal muscle	Age	0.4468	*p* = 0.0104	0.1–0.7
Relaxed masseter muscle	BMI	0.4292	*p* = 0.0142	0.1–0.7
Relaxed SCOM muscle	BMI	0.6445	*p* < 0.0001	0.4–0.8
Contracted SCOM muscle	BMI	0.5226	*p* = 0.0022	0.2–0.7
Relaxed masseter muscle	Height	0.4452	*p* = 0.0107	0.1–0.7
Relaxed masseter muscle	Weight	0.4997	*p* = 0.0036	0.2–0.7
Relaxed SCOM muscle	Weight	0.5945	*p* = 0.0003	0.3–0.7
Contracted SCOM muscle	Weight	0.4621	*p* = 0.0078	0.1–0.7

### Intra-Rater Reliability

In addition, the intra-reliability reported good reliability for fasciae (ICC_3,1_: 0.83; 0.69–0.89) and optimal for muscles (ICC_3,1_: 0.89; 0.85–0.92).

## Discussion

To the current knowledge, this study is the first that evaluates the thickness of the fasciae in the craniocervical district and compares it with the muscle thickness, the delta of muscle thickness, and the demographic characteristics. The fasciae of the head and neck serve as an important proprioceptive organ and are often involved in tension-type headaches, TMJ pain, acute and chronic neck, and shoulder pain, pain while chewing or swallowing, tinnitus, sinuses, vertigo, and vision, to mention a few (20). This study demonstrated that these fasciae are easily visualized appearing as linear, hyperechogenic layers (18), but with topographic peculiarities, which means that each muscular fascia has its proper mean thickness (*p* < 0.001), and we cannot generalize the data. With regard to the reliability, our data are consistent with other studies for the fasciae of other topographical regions (13–15) and for the head and neck muscles (7, 21, 22). Concerning the US fasciae thickness, there were no statistically significant differences between both sides (*p* > 0.05). This finding is consistent with other studies which assessed the US thickness of the deep/muscular fasciae in other districts (13–15), demonstrating that in healthy volunteers there were not differences statically significant between the two sides and highlighting significant differences among different levels/compartments of the same fascia or among different fascia as our study. In the future, it will be important to assess pathological patients, to understand if the craniocervical fasciae can change their thickness or asymmetric values could be detected. Differently with respect to the muscles, the deep fasciae do not change their thickness between the relaxed and contracted situations (*p* > 0.05). Really, it is important to keep in mind that the thickness of the fasciae and of the muscles is very different and that it is possible that also the fasciae may have thickness variations during muscular contraction, but these variations are under the possibility of the instrument to detect them.

In the present study, US thickness of the muscles varies between relaxed and contracted states, with a delta of muscle thickness of 3.28 ± 1.38 mm (right masseter), 2.93 ± 1 mm (left masseter), 1.72 ± 1.03 mm (right temporalis), 1.55 ± 1.06 mm (left temporalis), 2.43 ± 1.16 mm (right scom) and 1.97 ± 0.91 mm (left scom) (Table 2). Our findings are consistent with other studies that assessed the US muscle thickness in relaxed and contracted states (7). According to our results US muscles thickness, in the healthy volunteers, there were not statistically significant differences between both sides (*p* > 0.05), in both relaxed and contracted situations (*p* > 0.05), but if we analyzed each subject, it becomes evident that in 10 cases the muscles show a delta of muscle thickness that is reduced, with a prevalence of 38.46% among healthy volunteers. Interesting, in these cases often there is a misbalance between the temporal and masseter muscles, where a muscle is more able to contract on one side, and the other on the contralateral side. In such a way the motor tests are normal, but the correct movement is obtained using the temporalis and masseter muscles in a different way. This probably highlighted that these muscles act in coordination compensating each other to maintain excellent function. Indeed, the latter remains normal as demonstrated by a lack of statistically significant asymmetry. According to Pellagrama et al. (23), patients with TMJ dysfunction had greater muscle asymmetry than healthy individuals, and they also require greater muscle activity to execute stomatognathic movements and to keep the head posture.

The misbalance in the craniocervical muscles changes the movement strategy provoking a deteriorated function but compensated, with an overload that determines nociceptive impulses which imply the activation of non-coordinated stabilizing muscles of the cervical spine (4, 24). Consequently, there is increased activation of the superficial muscle such as SCOM which supports respiration exacerbating the functional misbalance. However, in relation to each muscle activation, the delta between the relaxed and contracted states was not statistically significant between both sides. The results obtained showed the symmetry between right and left (*p* > 0.05), even if the muscle activation is often asymmetric but not statistically significant (*p* > 0.05), though the delta of US muscle thickness had a huge range between different subjects: in the masseter muscle moves from the minimum value 0.5 mm to maximum value 4.2 mm, in the temporalis muscle from 0.1 to 3.9 mm and in the SCOM from 0.6 to 4.6 mm (Table 2). Various volunteers had a delta of muscle thickness values far from the mean (Table 2). Again, the differences of the delta of US muscle thickness between the two sides and among the various participants were not statistically significant (*p* > 0.05) (Table 3), however, evaluating this data for each subject, we found that in 10 subjects, the delta of thickness was less or more than 1 SD, demonstrating a deficit or an overactivity of muscle activation. Based on the previous studies and on the present study, the activation of different muscles in compensatory patterns, detectable with a US assessment can highlight postural adjustments used to maintain the functional activity that is not statistically significant if not contextualized within the individual postural and movements patterns. The individual alterations may be vulnerability factors that as Manfredini et al. (4) wrote: “even if anatomical factors may not be directly changed, the clinician can at least try to adapt/reduce the load or overuse, obviously considering psychosocial factors, which makes patients more vulnerable.” The findings of the present study did not intend to provide a cause or explanation for the development of TMJ dysfunction but want to underline the role of US imaging in a global evaluation of this relationship. Indeed, US imaging provides real-time static and dynamic assessment of the muscles as well as a more accessible and cost-effective option than other types of imaging (25, 26). Some authors used US imaging to assess and measure facial, masticatory, and neck muscles with good reliability (27, 28). Variables, such as probe pressure exerted on the underlying muscle, probe orientation, and muscle-site related to the absence of anatomical landmarks, may be significantly related to the different ultrasonographic techniques of having the patient maintain a slight interocclusal contact, clench, or maintain a physiologic rest position (4, 23, 29). Concerning such reasons, it is important to define clear evaluation protocols to analyze the muscles and fasciae of the craniocervical district, to permit a reliable US assessment. The present study demonstrated how the US imaging plays a crucial role in the assessment of the muscle strength by US thickness in relaxed/contracted states and with the calculation of the delta muscle thickness, permitting a better understanding of the participation of the masticatory muscles and cervical muscles in the performing the functions of the stomatognathic system. US may also be able to uncover functional changes which are invisible during the clinical inspection and unforeseen by current clinical practice. Finally, being able to define the specific functional disorders may facilitate a more targeted approach to treatment and prevent the onset of functional disorders as the initial phase of TMJ dysfunction.

### Limitations

The present study was developed in ultrasound B-Mode, not M-Mode or 4-D mode. In addition, color elastography may be useful to assess the fasciae of the head. Since the study involved only a small number of healthy volunteers and given the qualitative limitations of the US assessments, it was impossible for us to analyze the prevalence of the US findings or to make any hypotheses on causes, prognostic significance or therapeutic implications. Future longitudinal studies, such aslarger numbers of patients and healthy individuals will be able to contribute to our knowledge of the functional disorders before the pathophysiology of TMJ dysfunction. These studies should be performed with representative samples of patients with painful and non-painful TM disorders as well as with the non-TM disorders population.

## Conclusions

In conclusion, the results of this study corroborate that US imaging is a reliable tool to assess and measure the thickness of the fasciae and the muscles. Moreover, US imaging is a suitable tool to assess, identify functional asymmetry that could become symptomatic, allowing an early rehabilitation treatment. The delta of US muscle activation is an easy and fast parameter to use in daily practice.

## Data Availability Statement

The raw data supporting the conclusions of this article will be made available by the authors, without undue reservation.

## Ethics Statement

The studies involving human participants were reviewed and approved by University of Padua. Written informed consent to participate in this study was provided by the participants' legal guardian/next of kin.

## Author Contributions

CP, DG, VM, and CS conceived the study and co-wrote the paper. CP and CS extracted all data. CP, DG, VM, RDC, and CS undertook and refined the search. CP, CFe, CFa, VM, RDC, and CS helped to revise the intellectual content. All authors read and approved the final manuscript.

## Conflict of Interest

The authors declare that the research was conducted in the absence of any commercial or financial relationships that could be construed as a potential conflict of interest.

## Publisher's Note

All claims expressed in this article are solely those of the authors and do not necessarily represent those of their affiliated organizations, or those of the publisher, the editors and the reviewers. Any product that may be evaluated in this article, or claim that may be made by its manufacturer, is not guaranteed or endorsed by the publisher.
